# Assessment of Knowledge, Behaviour and Sun Protection Practices among Health Services Vocational School Students 

**DOI:** 10.3390/ijerph9072378

**Published:** 2012-07-04

**Authors:** Eray Yurtseven, Tumer Ulus, Suphi Vehid, Selçuk Köksal, Merve Bosat, Kutay Akkoyun

**Affiliations:** 1 Cerrahpasa Medical Faculty, Public Health Department, Istanbul University, 34320 Kocamustafa Pasa, Istanbul, Turkey; Email: tumerulus@gmail.com (T.U.); slckoksal@gmail.com (S.V.); cny@istanbul.edu.tr (S.K.); mervebosat@hotmail.com (M.B); 2 Department of Land Resources and Environmental Sciences, Montana State University, P.O. Box 173120, Bozeman, MT 59717, USA; Email: kutayakkoyun@gmail.com

**Keywords:** skin, cancer, sun, students, health

## Abstract

There has been a significant increase in the cases of skin cancer throughout the world in the last few decades. Although the mortality rate of skin cancer is relatively low, its impact on other health aspects is high and the treatment of undesired aesthetic damage is costly. According to disability-adjusted life year rates (DALY), 1.5 million days are estimated to be lost from people’s lives every year worldwide due to ultraviolet (UV) radiation. The purpose of this study was to raise sun health awareness levels among health services vocational school students. A total of 414 students were included in the analysis. A questionnaire form was used to evaluate knowledge, attitudes and behaviours among the survey sample. The average level of knowledge concerning the effects of the sun was found to be 8.64 ± 2.5 out of 15 points. All socio-demographic factors were analysed, but the only significant variables found were age and the possible presence of skin cancer within the immediate family (*p* < 0.05).

## 1. Introduction

Human exposure to solar ultraviolet radiation has important public health implications [1,2]. People are exposed to large quantities of UV radiation in part due to the thinning of the stratospheric ozone layer, but also through several other sources, such as: living and travelling in sunny climates, excessive sunbathing and sun bed use, outdoor sports, and the usage of appliances and devices that emit UV radiation in domestic and industrial settings [3,4,5]. There has been a significant increase in the incidence of skin cancer throughout the world in the last few decades, despite increasing medical awareness of the dangers of skin cancer and the advancement of diagnostic procedures [6]. Worldwide some 2–3 million cases of non-melanoma skin cancer and 132,000 cases of malignant melanoma skin cancer are diagnosed every year. Studies have found that 65-90% of the cases of melanoma skin cancer are caused by UV rays and it has been shown that in the USA one in five people develop skin cancer at some point in their lives [7,8,9]. Although the mortality rate of skin cancer is relatively low, its impact on other health aspects is high and the treatment of undesired aesthetic damage is costly. In terms of disability-adjusted life year rates (DALY), 1.5 million days are thought to be lost worldwide due to UV radiation. Today, one in three cancer cases are diagnosed as skin cancer [10]. According to 2011 epidemiology statistics from the cancer section of the Turkish Department of Health the incidence of skin cancer in Turkey is 18.91 per 100,000 population and it is the third most commonly diagnosed type of cancer [11]. Epidemiological studies show that the sun is the most significant environmental factor in regard to the development of skin cancer and many other skin conditions. The first step to reduce the incidence of skin cancer is to increase levels of awareness of the harmful effects of the sun and how one can better protect themselves from UV radiation [12,13]. It is vitally important that education reflect advances in knowledge in regard to protection against the potentially harmful effects of the sun so that changes in behaviour patterns are produced [12]. Education is the key in raising awareness [14,15]. Studies have been carried out in many countries to determine awareness levels of the effects of the sun, of skin cancer and of behaviour with regard to sun protection, usually with the intention of creating effective health campaigns to prevent skin cancer [5]. The purpose of this study is to raise levels of awareness, change attitudes and influence behaviour in regard to sun health among health services vocational school students.

## 2. Material and Methods

This cross sectional study was carried out between 15 September and 15 October 2011 on the Health Services Vocational School campus of Istanbul University. The study did not use sampling, as all 487 students on the campus were included. The questionnaire that was used for data collection was created through a search of relevant literature and consisted of eight questions about the socio-demographic characteristics of each student and 15 questions investigating the student’s knowledge of the effects of the sun [13,16,17]. Each correct answer equalled one point when calculating the average level of employee knowledge. Face to face interviews were conducted to collect data using the questionnaire. Subjects were given background information about the purpose of the study and its methodology and were given the opportunity to ask questions. Names are not used as they are not related to the reliability of the survey. Students who were absent or who did not wish to take part in the study have not been included. The data was analysed using SPSS (Statistical Package for Social Sciences) 15.0. Following the statistical evaluation of data and the summarization of frequencies and percentages, students t-test ANOVA and correlation analysis were used.

## 3. Results

The students who participated in the survey were aged between 18–30, with a mean age of 20.03 ± 2.37. Of the total 414 students who participated in the study 189 were male (45.7%) and 225 were female (54.3%). Of the sampled student’s parents, 164 (39.6%) had graduated from high school, and 40 (9.7%) were university graduates. Concerning the students’ family status, 337 (81.3%) were part of a typical nuclear family. One hundred and sixty four (164, 39.6%) said they had been sunburnt at some point in their lives and 19 (4.5%) were aware of someone in their family who had or who currently has skin cancer. The general levels of knowledge about the effects of the sun among students were 8.64 ± 2.35. All socio-demographic factors were analysed, but the only significant variables found were age and the possible presence of skin cancer within the immediate family (*p* < 0.05), none of the other factors had a significant effect (Table 1).

**Table 1:** Level of knowledge of students based on a variety of factors.

Variables	N	%	Mean level of knowledge ± SD	*p*
**Age**				
18–20	198	47.8	8.37 ± 2.87	*P* < 0.05
21–23	87	21.1	8.44 ± 2.24	
24–26	78	18.8	9.44 ± 2.04	
27–30	51	12.3	9.37 ± 2.23	
**Sex**				
Male	189	45.7	8.61 ± 2.5	*p* > 0.05
Female	225	54.3	8.67 ± 2.44	
**Parent’s education**				
Primary school leaver	55	13.4	8.37 ± 1.39	*p* > 0.05
Middle school leaver	19	4.5	9.17 ± 0.92	
High school leaver	164	39.6	8.66 ± 2.82	
University graduate	40	9.7	8.2 ± 1.9	
Masters level studies	118	28.4	9.21 ± 2.72	
Doctorate level studies	19	4.5	8.26 ± 3.84	
**Family**				
Nuclear	337	81.3	8.73 ± 2.75	*p* > 0.05
Extended	77	18.7	8.83 ± 1.63	
**Skin tone**				
Fair	180	43.3	8.44 ± 2.28	*p* > 0.05
Dark	234	56.7	8.9 ± 2.05	
**Having been sunburnt**				
Yes	164	39.6	8.85 ± 2.19	*p* > 0.05
No	250	60.4	8.77 ± 2.04	
**Having a close family member with skin cancer**				
Yes	19	4.5	9.95 ± 2.63	*p* < 0.05
No	395	95.5	8.58 ± 2.37	

Staying in the shade was cited as the preferred method of protection against the effects of the sun by 91.5% of the men and 95.5% of the women in the study. Using ‘an umbrella’ was found to be the least commonly used method of protection at 19.5% and 24.4% respectively (Table 2). 

**Table 2:** Students’ applied protection methods from sun light.

	Factor	Men		Women	
		N	%	N	%
**Sun protection measures taken**	Not going out between 10.00 (A.M) and 4.00 (P.M)	156	82.5	159	70.6
	Staying in the shade	173	91.5	215	95.5
	Wearing a hat	163	86.2	108	48.0
	Using an umbrella	37	19.5	55	24.4
	Wearing light colours	153	80.9	105	46.6
	Wearing light protective clothing	115	60.8	81	36.0
	Wearing sun glasses	111	58.7	197	87.5
	Using sun protection cream (SPC)	108	57.1	204	90.6

When the level of awareness of students regarding both positive and negative effects of the sun was investigated, the most well-known benefit proved to be that of synthesising vitamin D, with 73.7% of the men and 86.3% of the women citing it. The most well known negative effects of the sun for men and women was the appearance of age spots (75.4% and 94.5%, respectively), see Table 3. Table 3 also includes the participants’ source information of the effects of the sun, *i.e.*, how the participants had previously learned about the effects of the sun. TV at 78.6% and 76.7% was found to be the biggest source for both men and women in our research. 

**Table 3:** Students’ levels of knowledge about sunlight.

	Factor	Men		Women	
		N	%	N	%
**Beneficial effects of the sun**	Synthesis of vitamin D	139	73.7	194	86.3
	Treatment in some skin conditions	62	32.7	74	32.8
	Positive psychological effects	90	47.5	129	57.5
**Harmful effects of the sun **	Age spots	143	75.4	213	94.5
	Increased aging of the skin	99	52.4	136	60.5
	Skin cancer	133	70.4	157	69.8
	Sunburn	127	67.2	172	76.7
	Increased possibility of allergic reactions	121	63.9	191	84.9
**Sources of information about sunlight**	TV	148	78.6	172	76.7
	Internet	111	58.7	164	73
	Magazines / journals	108	57.3	135	60.2
	Friends / family	68	36.1	83	36.9
	Doctor	114	60.6	139	61.6

The questions which were answered correctly most often concerned the following information: “Exposure to the sun increases aging, wrinkling and discolouration of the skin”, which 89.8% of students knew to be true and “It is necessary to use sunscreen to avoid the harmful effects of exposure to the sun”, which 88.8% correctly identified as being true. The least understood factor concerned “Sunlight has an immunosuppressive effect’, which only 16.42% of students knew to be true (Table 4). Each correct answer was used in calculating the average score.

**Table 4:** A breakdown of student responses to questions (%).

	Questions about the effects of the sun	True	False	Don’t know
1	Sun bathing is damaging to health	**82.8**	3.7	13.5
2	Tanned skin does not protect from sunlight.	**67.2**	23.1	9.7
3	Vitamin D is absorbed from the sun through the skin	**79.1**	9.0	11.9
4	It is dangerous to stay in the sun for a long time with sunscreen	**82.1**	14.9	3.0
5	It is necessary to use sunscreen to avoid the harmful effects of exposure to the sun	**88.8**	3.7	7.5
6	Staying out of the sun for regular intervals do not to prevent sun burn	**25.4**	64.9	9.7
7	Sun burn can occur even when the sun on the skin does not feel warm	**58.2**	28.4	13.4
8	Exposure to the sun increases aging, wrinkling and discolouration of the skin	**89.8**	4.5	5.7
9	A tan is evidence of damage to the skin	**52.2**	39.6	8.2
10	It is possible to become sunburnt on a cloudy day	**53.0**	35.1	11.9
11	Sunlight is not beneficial behind the window glass	**38.8**	53.3	7.9
12	Sunburn in childhood is a more significant factor in skin cancer than sun burn as an adult	**68.7**	15.9	15.4
13	Sunlight has an immunosuppressive effect	**16.4**	52.2	31.4
14	Light coloured clothing is a better protection against the sun than dark coloured clothing	**86.6**	8.2	5.2
15	Loose clothes are better protection against the sun than tight clothes	**69.4**	22.4	8.2

## 4. Discussion

There has been a significant increase in the incidence of skin cancer over the last 20 years and it has been shown that the risk of someone developing the condition is related to cumulative sun exposure over a lifetime [18]. It has been suggested that around 80% of skin cancer cases are preventable with the implementation of sun protection measures and appropriate behaviours. In spite of this, the incidence of skin cancer is still rising [6]. In this study the most generally used method for avoiding the harmful effects of the sun was to stay in the shade. Ergin *et al.* reached a similar conclusion in their 2011 study [10]. While Kaymak *et al.* and Kokturk *et al.* found ‘not going out at peak times’ to be the most commonly adopted method of avoiding the harmful effects of the sun, with figures of 45.3% and 53.0% for men and women [13,19]. Our research found this method to be used by 82.5% of men and 70.6% of women. In international studies sun protective cream (SPC) was the most commonly used form of sun protection [18]. Our study showed that while 90.6% of women used sunscreen, only 57.1% of men did. The study done by Kaymak *et al.* found an average of 28.0% of men used SPC [13]. Kokturk *et al.* presented a figure of 46.3% for women using SPC [19]. Our research suggests that using an umbrella was the least adopted method of sun protection behaviour for men and women. Not going out in the sun between 10.00 (A.M) and 4.00 (P.M) was the method adopted by 82.5% of the men and 70.6% of the women in our study, although this was shown to be the most commonly used method in other studies [17,20]. It is possible that because our study was carried out during a school term with students who were indoors during the day that it did not occur to them that they might need this behaviour. Also in the summer in Turkey it is not common for people to use umbrellas for sun protection. In this respect, we recommend that use of umbrellas be taught from childhood. We established that for both men (75.4%) and women (94.5%) having already had age spots was seen as the most significant risk factor for future occurrences. Mikkilineni *et al.* had similar findings [21]. Today with increasing awareness of the subject, genetic predisposition appears to be seen as one of the main causes of skin cancer. A further important result that we were able to establish was that staying in the sun for a long time was seen to be a high risk factor. The analysis done by Uslu *et al.* showed that sunburns and an abundance of moles are also considered as high risk factors [17]. No significant differences were found in the level of awareness of students with regard to sex, the level of parent’s education, family type, skin tones or incidence of sunburns, although having an immediate family member with skin cancer did impact results. It is thought that families that have a history of skin cancer have a greater depth of knowledge about the subject since they havepersonally seen the effects of skin cancer. This is similar to results from the study by Ergin *et al.* 2011 [10].

Interestingly, as the study group age decreased, the average level of knowledge scores also decreased. This is a significant statistic (*p* < 0.05). This suggests that particularly among the students, as a result of education programs in schools, that in order to increase awareness of the harmful effects of the sun education is vital. All students, starting at primary school need to be informed about the harmful effects of the sun and appropriate sun protection behaviours. Lectures and seminars should also be provided in higher education. It is also recommended that information and training be provided to students at regular intervals within organisations.

In our research, we assessed the awareness of the benefits and dangers of the sun. It was found that 73.7% of the men were aware of the positive effect of vitamin D synthesis compared to 86.3% of the women. When it came to the least known effects of treatment in some skin conditions, these figures were 32.7% for men and 32.8% for women. In the study done by Kaymak *et al.* [13] in 2007 these figures were 82.7% and 75.8% for positive effects of sun, which bears some similarity to our results. In our study the most widely recognized negative effect of the sun proved to be age spots which were known about by 75.4% of men and 94.5% of women. One difference between our study and that of Kaymak *et al.* was that they were able to establish a degree of awareness of the sun being implicated in some allergic reactions. This is likely to be accounted for by differences in the populations surveyed. The most common sources of information about the effects of the sun were found to be TV and the internet. This suggests that increased levels of education and information with regard to the harmful effects of the sun through media channels could increase general awareness. It is recommended that legislation be put in place to increase the ease with which this information can be disseminated through the mass media to the general public. 

The most correctly answered question by the respondents at 89.8% was that of exposure to the sun causing the skin to age, wrinkle and discolour more rapidly. The least accurately understood premise, regarding that sunlight has an immunosuppressive effect, was only responded to correctly by 16.4% of the sample. In 2006 Uslu *et al.* obtained similar results in a study of doctors (95.4% and 15.3%, respectively) [17]. In 2001 Mikkilineni *et al.* proved that in several countries as little as 1 or 2 hours of training was enough for some skin cancers to become recognizable to students and for some preventative behaviours to be adopted [21]. However, further investigation of the data suggested that regardless of the level of education some erroneous beliefs continued to exist and that the general public needed to be better informed on some subjects. 

## 5. Conclusions

In conclusion, in Mediterranean countries like Turkey, having a tanned complexion in the early part of the summer is seen as cosmetically desirable. At the same time, melanoma and other skin cancers are being seen more frequently, although due to the lack of comprehensive records, full and accurate statistics are not available [2,9]. This means that neither the research into the subject nor general health warnings are sufficient. This study highlights the need for the mass media to be used to increase the adoption of sun protection behaviours such as wearing protective clothing and routinely using sun screen among the general public. It is also suggested that open air sporting events and general entertainment activities be scheduled at safer times with the hopes of decreasing excessive sun exposure. And finally, the implementation of general education programmes that lead to life-long, sun-safe behaviours is greatly encouraged.
